# Investigation on the electronic structure, optical, elastic, mechanical, thermodynamic and thermoelectric properties of wide band gap semiconductor double perovskite Ba_2_InTaO_6_

**DOI:** 10.1039/c9ra00313d

**Published:** 2019-03-26

**Authors:** Sajad Ahmad Dar, Ramesh Sharma, Vipul Srivastava, Umesh Kumar Sakalle

**Affiliations:** Department of Physics, Govt. Motilal Vigyan Mahavidyalaya College Bhopal-462008 Madhya Pradesh State India sajad54453@gmail.com; Department of Applied Science, Feroze Gandhi Institute of Engineering and Technology Raebareli-229001 Uttar Pradesh State India; Department of Physics, School of Chemical Engineering & Physical Sciences, Lovely Professional University Phagwara-144411 Punjab State India; Department of Physics, Govt. PG College, BHEL Bhopal-462022 Madhya Pradesh State India

## Abstract

In the present paper, double perovskite Ba_2_InTaO_6_ was investigated in terms of its structural, electronic, optical, elastic, mechanical, thermodynamic and thermoelectric properties using density-functional theory (DFT). The generalized gradient approximation (GGA) in the scheme of Perdew, Burke and Ernzerhof (PBE) and the modified Becke–Johnson (mBJ) potential were employed for the exchange–correlation potential. The computed lattice constant was found to be in agreement with the available experimental and theoretical results. The electronic profile shows a semiconducting nature. Further analysis of the complex dielectric constant *ε*(*ω*), refractive index *n*(*ω*), reflectivity *R*(*ω*), absorption coefficient *α*(*ω*), optical conductivity (*ω*) and energy loss function were also reported with the incident photon energy. The elastic constants were also calculated and used to determine mechanical properties like Young's modulus (*Y*), the shear modulus (*G*), Poisson's ratio (*ν*) and the anisotropic factor (*A*). The electrical conductivity (*σ*/*τ*) and Seebeck coefficient (*S*) also demonstrated the semiconducting nature of the compound with electrons as the majority carriers. The value of the power factor was calculated to be 1.20 × 10^12^ W K^−2^ m^−1^ s^−1^ at 1000 K. From thermodynamic investigations, the heat capacity and Grüneisen parameter were also predicted.

## Abstract 2

In the present paper, double perovskite Ba_2_InTaO_6_ was investigated in terms of its structural, electronic, optical, elastic, mechanical, thermodynamic and thermoelectric properties using density-functional theory (DFT).

## Graphical abstract

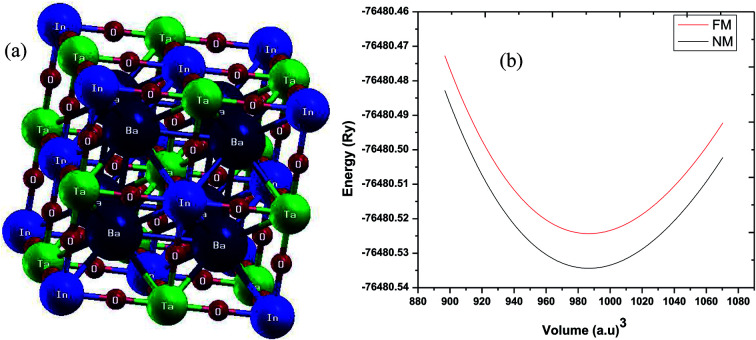

## 1. Introduction

Perovskites are a class of materials with rich diversity and have hence attracted researchers from all over the globe in the last decade. These materials are employed for novel applications like solid oxide fuel cells, perovskite fuel cells, spintronics applications, *etc.* Among these innovative materials, double perovskites have developed an important role. Double perovskite oxides have been widely utilized in the fields of thermoelectricity, solar energy conversion, transparent conductors, heterogeneous catalysts, pigments and photocatalysts. Double perovskite compounds were discovered in the early 1950s^1^ and they have remarkable properties and industrial applications.^2–9^ Advancements in computing power along with improvements in quantum modeling allow one to perform effective and precise quantum mechanical calculations and have thus overextended the calculating power to such an extent that those properties of materials which once looked impossible to determine can now be easily calculated with great precision.^10–13^ The present scenario with escalation in the industries and the increasing demand for natural resources to overcome the energy demands forces us to not only search for alternative energy sources but also reduce our energy consumption levels. Thermoelectric materials have received worldwide attention in their ability to convert waste heat into useful energy and hence are seen as forthcoming sources of green energy.^14–17^ At present, the world is in need of thermoelectric materials to decrease the dependence on vanishing fossil fuels. Perovskite materials with quite pleasing thermoelectric performance are represented as promising aspirants for thermoelectric devices.^18–24^ In recent years, double perovskites have received great attention as far as high-temperature thermoelectric investigations are concerned.^25–27^ Wide band gap materials have also been given attention for their high-temperature thermoelectric properties,^28^ and further, double perovskites have also been investigated for their electronic, thermoelectric, optical and magnetic properties using DFT.^29–31^

Ba_2_InTaO_6_, an important member of the vast perovskite family, has not yet been investigated for its structural, elastic, mechanical, thermodynamic and thermoelectric properties. Lufaso *et al.*^32^ have investigated Ba_2_InTaO_6_ and found it to crystallize in an ordered cubic double perovskite structure with a lattice parameter of 8.2814 Å. Dutta *et al.*^33^ have theoretically investigated Ba_2_InTaO_6_ with respect to its electronic and optical properties. Hammink *et al.*^34^ have investigated Ba_2_InTaO_6_ under a different range of temperatures. They observed that the material has cubic symmetry, space group *Fm*3̄*m* (225), a lattice constant of 8.282 Å with Ba atoms at 8c (1/4, 1/4, 1/4), In atoms at 4b (0.5, 0.5, 0.5), Ta cited at 4a (0, 0, 0) and O atoms at position 24e (*x*, 0, 0) of the unit cell where *x* = 0.2565. Zhou *et al.*^35^ have reported structural phase transition in Sr-doped Ba_2_InTaO_6_, using synchrotron X-ray and neutron powder diffraction techniques, where Ba_2−*x*_Sr_*x*_InTaO_6_ underwent a series of phase transitions from cubic to monoclinic. Song *et al.*^36^ have also reported the ordered cubic structure in Ba_2_InTaO_6_. They further observed it as a wide band gap semiconductor with photocatalytic activity for overall water splitting under UV irradiation. Hence, as far as the literature is concerned, a systematic study on this compound within the most successful density functional theory (DFT)^37,38^ is absent.

Therefore, in the present work, an attempt has been made to investigate its electronic, elastic, mechanical, optical, thermoelectronic and thermodynamic properties within GGA^39^ and mBJ.^40^ The Quasi-harmonic Debye model^41–43^ was used for the investigation of pressure- and temperature-dependent thermodynamic behavior including specific heat, bulks modulus, volume, Grüneisen parameter, *etc.* Post-DFT Boltztrap code^44^ was employed for thermoelectric investigation. Therefore, the present investigation may provide important data for future experimental and theoretical investigations.

## 2. Computational methodology

To investigate the double perovskite Ba_2_InTaO_6_ with respect to its electronic, mechanical, thermal and thermoelectric properties, the full-potential linearized augmented plane-wave method (FP-LAPW)^45^ based upon density functional theory (DFT) as implemented in WIEN2K code^37,38^ was used. It has been found that Ba_2_InTaO_6_ crystallizes in the simple B1-phase (NaCl-type structure).^32,34,36^ In the unit cell, the position of 8c (0.25, 0.25, 0.25) is occupied by Ba atoms, Ta at 4b (0.5, 0.5, 0.5), In at 4a (0, 0, 0) and 24e (*x*, 0, 0) by O atoms where *x* = 0.2565.^34^ Optimization of the structure was achieved using generalized gradient approximation (GGA) scheme of Perdew, Burke and Ernzerhof (PBE),^39^ considering the ferromagnetic and paramagnetic phases. Furthermore, the electronic properties were also investigated within the modified Becke–Johnson (mBJ).^40^ For the convergence of energy, the multiplication of the atomic radius, *R*_MT_ and **k**, vector was set to 7 (*R*_MT_*K*_max_ = 7), the angular moment *L*_max_ was set to 10, and *G*_max_ = 12 (a.u.)^−1^. To avoid any leakage of charge from the core, we used the *R*_MT_ (muffin-tin radii) values of 2.60, 2.12, 2.10 and 1.63 for Ba, In, Ta and O, respectively. The convergence was considered when the total energy was stable within 0.001 Ry and the charge difference was less than 0.001e per a.u.^3^ per unit cell. The tetrahedron method^46^ was used to calculate the density of states with a mesh of 1000 *K* points in Brillouin-zone integration. Further, the Charpin method^47^ as integrated into the Wien2k package was used for elastic constant calculations. Furthermore, various thermodynamic parameters were generated using the Quasi-harmonic Debye model^41–43^ while tuning temperature and pressure.

## 3. Results and discussion

### 3.1 Structural properties and electronic investigation

**Fig. 1:**
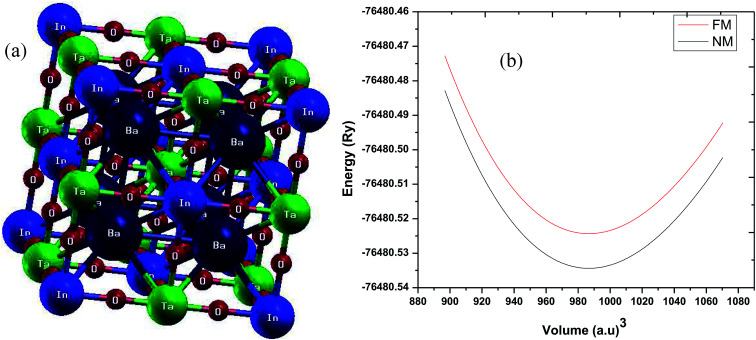
(a) Crystal structure of Ba_2_InTaO_6_; (b) calculated total energy as a function of volume for ferromagnetic (FM) and non-magnetic (NM) phases for Ba_2_InTaO_6_.

As stated above, the structure of Ba_2_InTaO_6_ is NaCl-type with an experimental lattice parameter value of 8.282 Å.^34^ The structural optimization based on Murnaghan's equation of state^48^ was performed to obtain a relaxed structure with minimum energy in the paramagnetic (PM) and ferromagnetic (FM) states. The obtained ground structure results including lattice parameters are summarized in Table 1. Our calculated values of the lattice parameters are slightly higher than the available experimental data. This is usually expected for GGA as it tends to overestimate the lattice parameters. The crystal structure and the optimization of Ba_2_InTaO_6_ were presented in Fig. 1(a and b), respectively. It has been found, as depicted in Fig. 1(b), that the *E*–*V* curves show a minimum energy in the paramagnetic (PM) state, and hence Ba_2_InTaO_6_ is stable in the PM state. The optimized ground state properties like the lattice constant (*a*_0_), bulk modulus (*B*_0_), and the pressure derivative of the bulk modulus (*B*′) were found to be very close to the available experimental and other theoretical data, which are summarized in Table 1. We have also calculated the bond lengths between Ba–O, In–O and Ta–O and when compared with the available experimental results^34^ as presented in Table 1, a good agreement is found.

**Table 1:** Lattice constant (Å), unit cell volume (a.u)^3^, bulk modulus (*B* in GPa), derivative of the bulk modulus (*B*′), ground-state energy (*E*_0_ inRy) and bond lengths (Å) calculated and compared with the experimental and theoretical values of Ba_2_InTaO_6_

Ba_2_InTaO_6_	Present	Other
Lattice constant	8.362	8.28 (ref. 32)
		8.70 (ref. 33)
		8.28 (ref. 34)
		8.29 (ref. 36)
Volume	986.67	—
*B*	155	142 (ref. 33)
*B*′	4.58	4.64(ref. 33)
Ba–O	2.93	2.92 (ref. 34)
Δ*E*_H_(kJ mol^−1^)	−2626.72	
In–O	2.13	2.11 (ref. 34)
Ta–O	2.03	2.02 (ref. 34)
Bandgap		
GGA (eV)	4.0	4.17 (ref. 36)
		3.82 (ref. 49)
mBJ (eV)	5.0	

To further verify the thermodynamic stability, we computed the enthalpy of formation (Δ*H*) by taking the energy difference between the compound and its individual atoms from the following equation:Δ*H* = *E*_total_(Ba_2_InTaO_6_) − *aE*_Ba_ − *bE*_In_ − *cE*_Ta_ − *dE*_O_Here, *E*_total_(Ba_2_InTaO_6_) is the total energy of Ba_2_InTaO_6_ and *E*_Ba_, *E*_In_, *E*_Ta_, *E*_O_ are the energies of the discrete atoms in their intrinsic lattice, per unit cell volume. The number of atoms of Ba/In/Ta/and O per unit cell volume are represented by the letters *a*, *b*, *c* and *d*, respectively. The calculated formation energies of Ba_2_InTaO_6_ is given in Table 1.

**Fig. 2:**
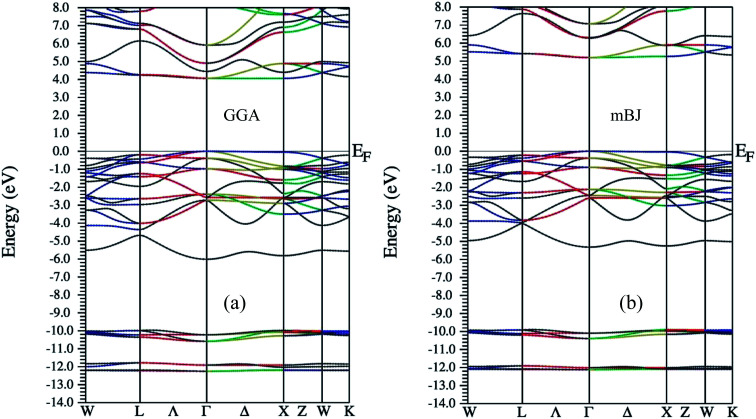
Band structure for Ba_2_InTaO_6_ within (a) GGA (b) mBJ.

As far as electronic structure calculation of Ba_2_InTaO_6_ is concerned, it has been calculated along the high symmetry directions using GGA and mBJ in Fig. 2. Fig. 2(a and b) present the diagram of the band structures within GGA and mBJ exchange–correlation potentials, respectively. It is clear from the figures that the material presents semiconducting nature in both approximations. In GGA, the calculated value of the band gap is high and estimated to be 4.0 eV, while in mBJ it is somewhat higher (*i.e.*, 5.15 eV). Lv *et al.*^49^ have reported Ba_2_InTaO_6_ as an important candidate for photocatalytic properties with a direct band gap observed at around 3.82 eV. So utilizing the mBJ potential, the band gap is overestimated. From the examination of band structure utilizing the PBE exchange–correlation potential, we see that it is a direct band gap semiconductor with a band gap of 4.0 eV, which is closer to the experimental value.^49^ The valance band maxima (VBM) and the conduction band minima (CBM) were found to lie on same symmetry point ‘Γ’ in both approximations, hence presenting the nature of the direct band gap. The direct band gap of both approximations with other theoretical and experimental results are listed in Table 1.

**Fig. 3:**
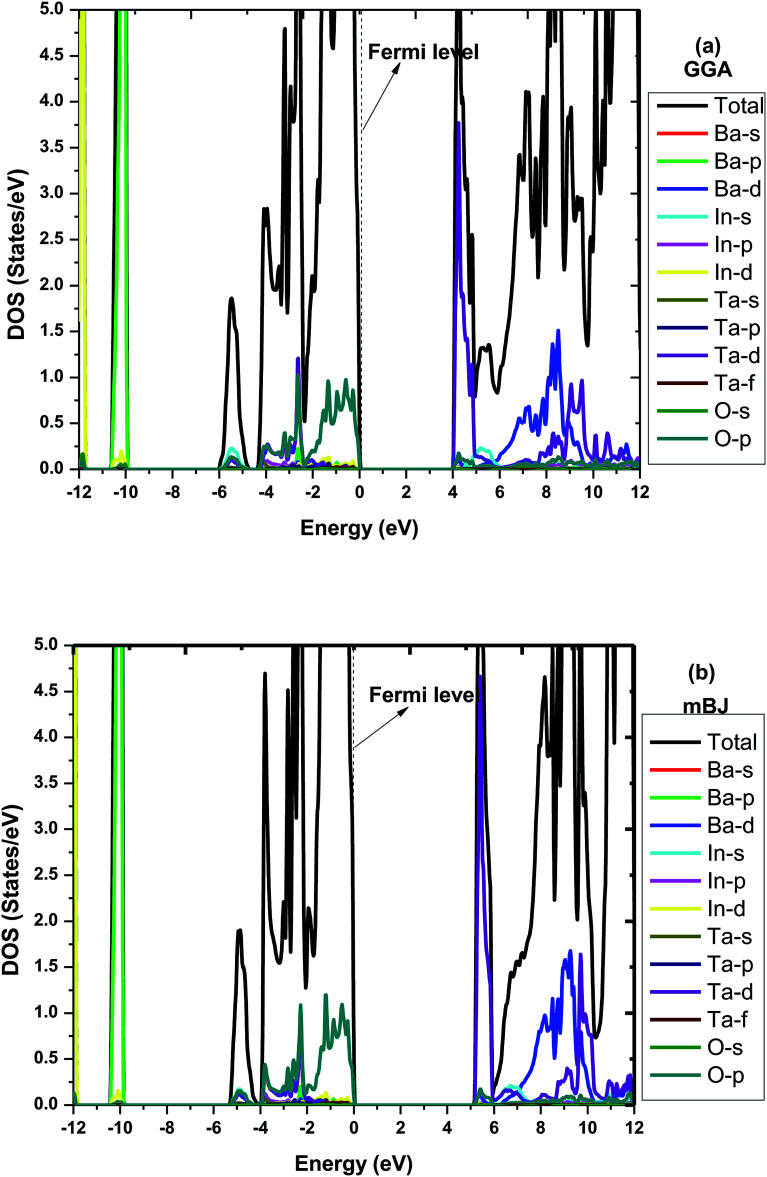
Combined total and partial density of states for Ba_2_InTaO_6_ within (a) GGA (b) mBJ.

To make it more clear, we have plotted the combined total density of states (TDOS) and partial density of states (PDOS) within GGA and mBJ in the range of −12 to 12 eV as shown in Fig. 3(a and b), respectively. Both figures represent a wide band gap semiconducting nature. Here in these plots, we can also understand the elemental contribution to TDOS. The density of states contour indicates that the upper region of the valence band extending from 0 to −5 eV is predominantly occupied due to O p states with a small aggregate of Ta ‘d’ states, while the bands in the energy range of −10.3 eV to −10.0 eV are due to the Ba ‘p’ states. In the conduction band, the Ta ‘d’ states are responsible for the bands in the region of 2.5 eV to 4.8 eV. The bands occurring inside the higher energy level of the conduction band are due to the hybridization between the In ‘d’ states with a small aggregate of Ba and Ta ‘d’ states. The main contribution to the valance band in both GGA and mBJ is found from the Ba-p, In-d and O-p states, while for the conduction band, Ba-d and Ta-d are dominant and traces of In-s were also noticed. One can observe from the DOS plots that hybridization between the In-d and O-p states is present in the valance band close to the Fermi-level. These bands close to the Fermi-level were observed for both GGA and mBJ. The bands due to the Ba-d, Ta-d and O-p states present in the conduction band were observed to change with the application of mBJ. In case of GGA, these bands are present from 3.9 eV, hybridized with one another; however, in case of mBJ, these bands were found to shift towards a higher energy conduction band and were found at 4.5 eV, hence increasing the band gap. Therefore, both band structure and DOS show the wide band gap nature of the material.

### 3.2 Elastic and mechanical properties

Elastic and mechanical properties of materials, particularly manufacturing materials become important, where materials are subjected to forces both during fabrication and processing. In this context, it becomes necessary to know how atom-to-atom bonding and further mechanical stability can also be defined. We have, therefore, investigated elastic constants (*C*_11_, *C*_12_, *C*_44_) under ambient conditions for Ba_2_InTaO_6_. The elastic constants were calculated by the Thomas Charpin method.^47^ The existence of the crystal isn't possible in the stable or meta-stable state unless its elastic constants obey the generalized mechanical stability criteria.^50^ Our calculated values of the elastic constants and Bulk moduli properly follow the cubic stability criteria. From the values of the elastic constants, mechanical properties like Young's modulus (*Y*), which presents the strength of the material; the bulk modulus (*B*), which describes the stiffness of a material, higher the value of B, the higher its stiffness resistance is; and the shear modulus (*G*), which describes the calculated plastic twist of a material. The value of the shear modulus (*G*) has been obtained from the Voigt–Reuss–Hill approximation^51,52^ using *G*_V_ and *G*_R_ and is presented in Table 2. One can find the physical formula to calculate *G*_V_ and *G*_R_ in ref. 42. The obtained value of the shear modulus is 88.24 GPa, which is small, meaning that the compound shows less plastic twist.

**Table 2:** Calculated elastic constants *C*_11_, *C*_12_, *C*_44_ in (GPa), the bulk modulus *B* (GPa), the shear modulus *G* (GPa), Young's modulus *Y* (GPa), Poisson's ratio *ν*, the Zener anisotropy factor *A*, the *B*/*G* ratio, Cauchy pressure *C*_12_–*C*_44_ and the melting temperature *T*_m_ (K) for Ba_2_InTaO_6_ under ambient conditions

GGA	Ba_2_InTaO_6_
*C*_11_	282.67
*C*_12_	91.39
*C*_44_	85.22
*B*	155
*G*_V_	89.38
*G*_R_	89.10
*G*	89.24
*Y*	224.64
*ν*	0.2587
*B*/*G*	1.73
*C*_12_–*C*_44_	6.13
*A*	0.891
*T*_m_	2223 ± 300

The value of Young's modulus (*Y*) was obtained from the bulk modulus (*B*) and the shear modulus (*G*).^42^ As *Y* deals with the strength of the material, the higher the value of *Y*, the higher its strength is. The obtained value of *Y* was calculated to be 224.64 GPa, which is large enough, and hence, Ba_2_InTaO_6_ behaves as a hard material. Another parameter anisotropic factor *A*^42,53^ was also calculated and found to be 0.891 (less than 1), signifying that the material has elastic anisotropic nature.

In order to define the ductile or brittle behavior of Ba_2_InTaO_6_, we obtained the *B*/*G* ratio, which was calculated to be 1.73, and according to Pugh,^54^ the *B*/*G* ratio is near to 1.75 in value and shows a ductile nature. An estimation of ductility and brittleness can also be obtained from the Cauchy pressure value (*C*_12_–*C*_44_); positive values characterize a material as ductile and negative values as brittle. From the information in Table 2, it is clear that the Cauchy's pressure is positive for Ba_2_InTaO_6_, which emphasize its ductile nature. Poisson's ratio ‘*ν*’ is another rule utilized for determining the ductility and brittleness of a compound. If its value is greater than 0.26, the material possesses ductile nature; otherwise, the material is considered to be brittle. Table 2 shows that Poisson's ratio for Ba_2_InTaO_6_ is close to 0.26, which confirms its ductile nature. Poisson's ratio ‘*ν*’ also gives knowledge about the bonding forces available in solid materials. For central force solids, Poisson's ratio has a range of 0.25 to 0.5.^55^ From the data in Table 2, it is clear that our computed Poisson's ratio lies in this range, implying that the bonding forces are of central types. As per our insight, no theoretical or experimental data exist in the literature on this compound for elastic properties to which we can compare our work. We believe that our work can stimulate further research in this direction in the future.

We have also obtained the melting temperature of the compound^56^ equal to 2223 ± 300 K under ambient conditions from the elastic constant values by using the following equation:*T*_m_ (K) =[553 (K) + (5.911)*C*_12_] GPa ± 300 K

The computed values of the elastic constants, mechanical properties and melting temperature are grouped in Table 2.

### 3.3 Optical properties

Motivated by the prospect of using its interesting electronic structure for optoelectronic semiconductor applications, the optical and electronic transport properties of the double perovskite oxide Ba_2_InTaO_6_ were studied. The frequency-dependent optical properties were studied and calculated through the dielectric functions.^57,58^ It was realized that the optical properties of a solid can be portrayed by the complex dielectric work *ε*(*ω*), which has two sections, real and imaginary, and can be communicated as:*ε* = *ε*_1_(*ω*) + i*ε*_2_(*ω*)

The imaginary part *ε*_2_(*ω*), which arises from interband and intraband transitions, shows the possible transitions from the occupied to unoccupied states with fixed (**k**-vectors) over the Brillouin zone (BZ), which are dependent on density of states (DOS) and the momentum matrix *P*; it can be mathematically defined as:
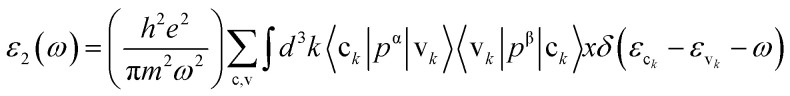
where *p* represents the moment matric element between the states of band α and β within the crystal momentum *k*, while c_*k*_ and v_*k*_ are the crystal wave functions corresponding to the conduction and valence bands with the crystal wave vector *k* from the imaginary part using the Kramers–Kronig relationship, which provides the real part *ε*_1_(*ω*) of the dielectric function:^57^
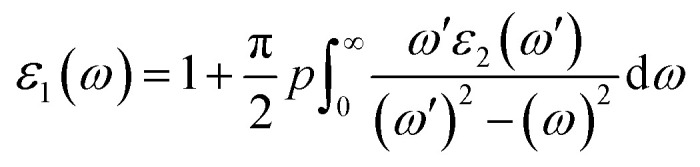
where *p* implies the principal value of the integral.

The reflectance can be calculated using the relation:
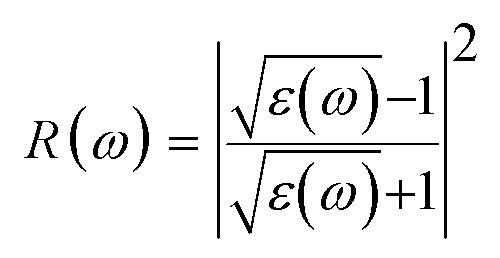


The absorption coefficient (*α*) can be deduced from the dielectric function as given below:
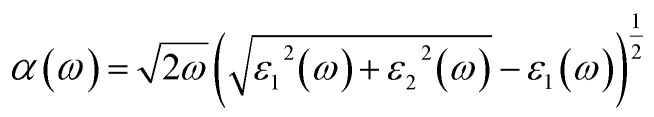


The real and imaginary parts of the dielectric function allow us to obtain some important optical parameter like the refractive index *n*(*ω*) and the extinction coefficient *k*(*ω*).
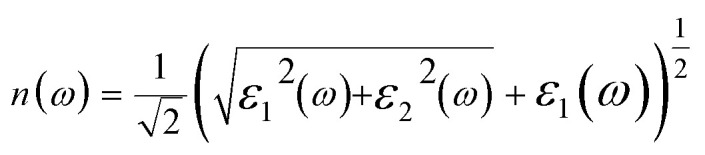

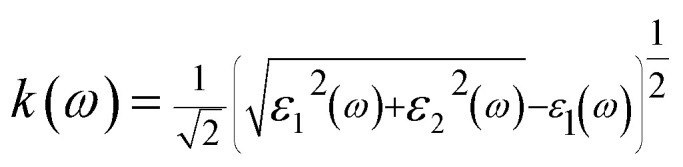


**Fig. 4:**
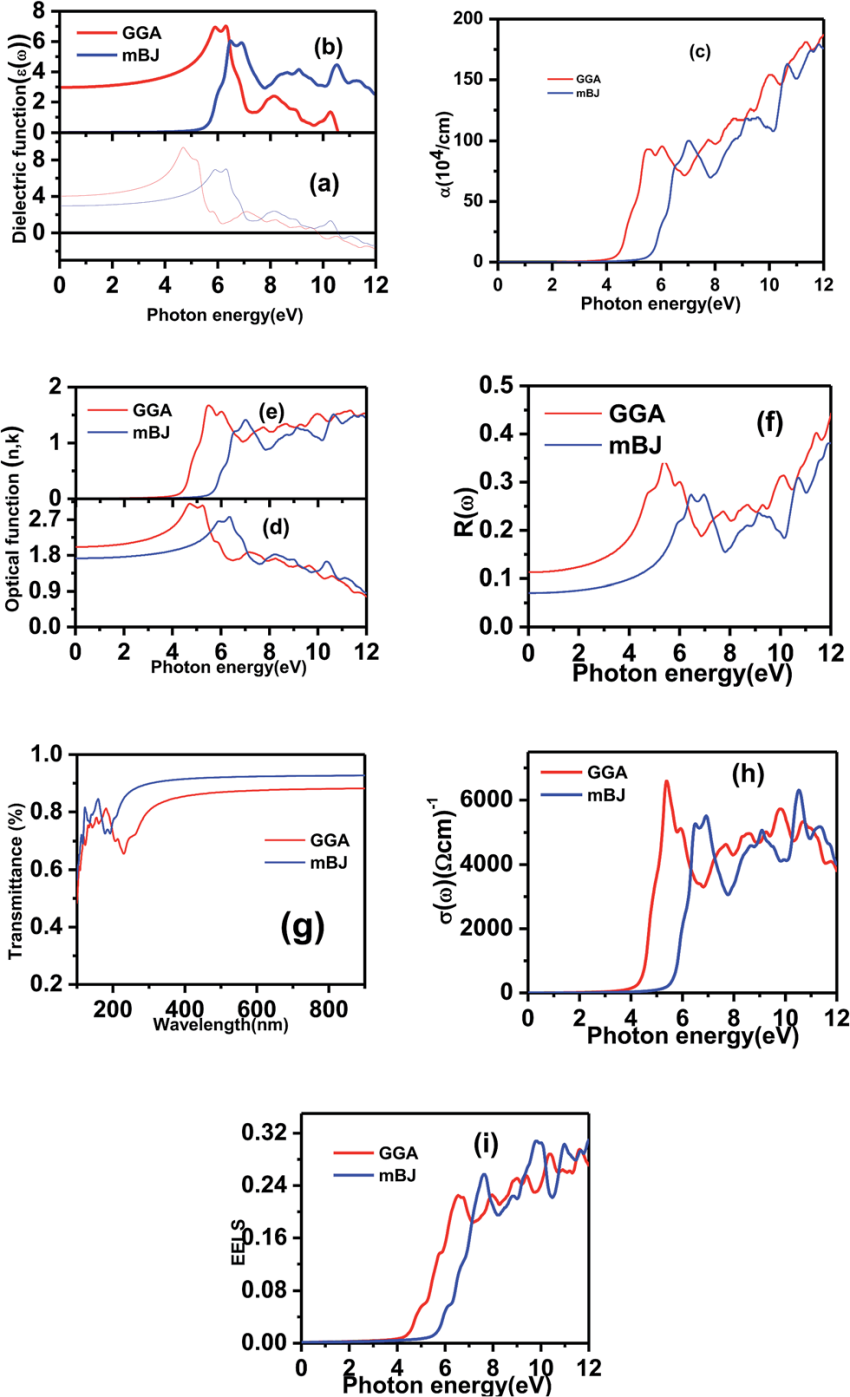
Optical spectrum of the dielectric constant as a function of the photon energy of Ba_2_InTaO_6_ within (a) GGA (b) mBJ. (c) Calculated absorption spectrum of Ba_2_InTaO_6_ within (a) GGA (b) mBJ. (d) and (e) Refractive index spectrum of Ba_2_InTaO_6_ within (a) GGA (b) mBJ. (f) Reflectivity spectrum of Ba_2_InTaO_6_ within (a) GGA (b) mBJ. (g) Transmittance spectrum of Ba_2_InTaO_6_ within (a) GGA (b) mBJ. (h) Optical conductivity spectrum of Ba_2_InTaO_6_ within (a) GGA (b) mBJ. (i) Optical spectrum of the electron loss spectrum as a function of the photon energy of Ba_2_InTaO_6_ within GGA and mBJ.

In this work, we focused on the optical parameters such as dielectric function, absorption coefficient *α*(*ω*), energy loss spectrum *L*(*ω*), reflectivity *R*(*ω*) and refractive coefficient, which can be obtained from *ε*_1_(*ω*) and *ε*_2_(*ω*). The optical calculations of Ba_2_InTaO_6_ were presented in Fig. 4(a–i) as follows: Fig. 4(a) and (b) for the dielectric function, Fig. 4(c) for the absorption spectra, Fig. 4(d and e) for the refractive coefficient, Fig. 4(f) for reflectivity, Fig. 4(g) for the transmittance spectra, Fig. 4(h) for optical conductivity and Fig. 4(i) for the energy loss function.

The compound under investigation Ba_2_InTaO_6_ has cubic symmetry. We have the principal tensor component *ε*_*xx*_ = *ε*_*yy*_ = *ε*_*zz*_ for cubic symmetry; therefore, we have calculated only one component of the complex dielectric function. As shown in Fig. 4(a), static dielectric constants *ε*_1_(0) of Ba_2_InTaO_6_ are 4.07 (GGA), 2.97 (mBJ). After reaching maximum values, it starts to decrease and flattens with few variations. In the energy range of 0–9.8 eV (GGA), 0–10.57 eV (mBJ), *ε*_1_(*ω*) is shown to be positive due to the semiconductor nature, while in the energy range of 9.8 to 12 eV (GGA), 10.57 to 12 (mBJ), the *ε*_1_(*ω*) value is negative, which shows the characteristics of the metal due to the transition of the Ta-5d electrons.

The imaginary part *ε*_2_(*ω*) of the dielectric function in Fig. 4(b) shows the principal peak of the optical critical point at 7.06 (GGA), 6.09 (mBJ), with the corresponding energy range 6.30 eV (GGA), 6.47 eV (mBJ) arising due to the transition between some noticeable peaks located at 5.90 eV, 8.15 eV, and 10.26 eV (GGA); for mBJ, these peaks are located at 6.90 eV, 9.07 eV, and 10.50 eV. The threshold point of optical direct transition corresponding to the *X*_v_ − *X*_c_ splitting (among the lowest conduction band and the highest valence band) is recognized as the absorption edge. Above this threshold point, with the increase in energy, we have seen that the curve increases instantaneously due to the real part *ε*_2_(*ω*) of the dielectric function. The transition between the occupied states of the valence region to the unoccupied states of the conduction region arise due to rapid peak in the optical spectrum. The density of states defined by the origin of these peaks lies in the interband transition. For mBJ as compared to GGA, the principal peak of the real and imaginary parts shifts due to the increase in energy.

In Fig. 4(c), we have shown the absorption coefficient, which is represented as the attenuation percentage of light intensity that propagates per unit distance in a material. The shape of the absorption spectrum is quite similar to the peak of the imaginary part *ε*_2_(*ω*) of the complex dielectric function, showing that through the linear absorption spectra originating from the imaginary parts of the electronic dielectric function, we found peaks at the energies of 4.5 eV (GGA), 5.7 eV (mBJ); 5.1 eV (GGA), 7.02 eV (mBJ); 6.8 eV (GGA), 7.9 eV (mBJ) and 7.8 eV (GGA), 8.6 eV (mBJ). At the lower energy range from 0 to 4 eV, there is no absorption, meaning that the material is transparent from the partially ultra-violet to the visible light area due to the phonon energy within the forbidden band. The absorption region is more stretched towards high energy for mBJ as compared to GGA. As we can see from the absorption spectra, as the energy decreases, the absorption coefficient decreases rapidly, which shows the behavior of common semiconductors. In general, the differences among the propagation of an electromagnetic wave *via* vacuum and some different material may be described as *N*(*ω*) = *n*(*ω*) + i*k*(*ω*). The computed refractive index profile and the extinction coefficient of Ba_2_InTaO_6_ are displayed in Fig. 4(d). As can be seen, the refractive index increases with increasing energy and reaches a maximum. The same can be observed for the dielectric characteristic function, and these are associated with the relation *n*(0) = √*ε*(0); the static refractive index *n*(0) was found to have the value 2.01 (0.05 eV) (GGA), 1.72 (0.043 eV) (mBJ) as can be seen from Fig. 4(d). The same value was calculated *via n*(0) = √*ε*(0) = 2.0 (GGA), 1.72 (mBJ). The principal peak of the refractive index occurs at 3.12 (4.70 eV) for GGA, 2.76 (6.35 eV) for mBJ. The nature of the refractive index and dielectric function are both the same. The variation of the extinction coefficient *k*(*ω*) as a function of photon energy is shown in Fig. 4(e). From the extinction coefficient *k*(*ω*) spectrum, we determined that the *k*(*ω*) of Ba_2_InTaO_6_ increases with energy in the transparent region and reaches a maximum value. The *k*(*ω*) was found to have the highest value at 5.48 eV (GGA), 1.52 eV (mBJ), which indicated that absorption is also notable at about 5.48 eV (GGA), 1.52 eV (mBJ).

The computed reflectivity spectrum *R*(*ω*) as shown in Fig. 4(f) shows that the zero frequency limit of reflectivity *R*(*ω*) was found to be 0.11 for (GGA) and 0.07 for (mBJ). The most illustrated reflection peaks at energies for GGA are 5.39 eV, 7.73 eV, 8.70 eV, 10.08 eV, 11.48 eV and for mBJ are 6.45 eV, 6.95 eV, 9.11 eV, 10.70 eV corresponding to the negative values of *ε*_1_(*ω*), which shows that there is a highest value of reflectivity due to the interband transition. The plot of the transmittance *versus* wavelength is presented in Fig. 4(g); as can be seen from the figure, Ba_2_InTaO_6_ has high transparency in the visible and IR region due to the wide band gap, while the transmittance has a lower value. Considerable variation arises from the enlarged optical band gap and reflectivity in the UV region. The transmittance of GGA becomes larger than the mBJ in the UV region; however, for IR and the visible light region, mBJ shows a larger transmittance than GGA. The average optical transmittance of Ba_2_InTaO_6_ is 86% (GGA) and 91% (mBJ), respectively. Thus, this double perovskite Ba_2_InTaO_6_ in its structure acts transparently in the visible light region and absorbent in the ultraviolet region.

The calculated real part of the optical conductivity profile is depicted in Fig. 4(h). As shown in the figure, it can be seen that the first transition located at the energies of 6617.26 (5.34 eV), 5300.68 (6.48 eV) for GGA and mBJ, respectively, arises in the *Γ* direction of the Brillouin zone. The second transition occurs from an sp transition. The electron energy-loss function (ELF) defines interactions such as interband, intraband and plasmon. The energy which is lost by a fast-moving electron while travelling through the material is ELF. The ELF is computed and depicted in Fig. 4(i). It can be seen that the maximum energy loss function *L*(*ω*) with GGA occurs at 0.29 (11.66), whereas for mBJ, the maximum energy loss function occurs at 0.31 (9.75 eV); the abrupt reduction of reflectivity spectrum arises due to the sharp peak of the energy loss spectrum. There is no loss in the region from 0 to 4 eV (GGA) and 0 to 5 eV (mBJ). The computed results for the optical properties of Ba_2_InTaO_6_ are in accordance with the results of the optical properties measured by Zurmuhlen *et al.*^59^

The computed value of the real part of the dielectric function *ε*(0), the refractive index *n*(0), the extinction coefficient *k*(*ω*), reflectivity *R*(0), the optical absorption coefficient *α*(*ω*), the real part of optical conductivity *σ*(*ω*) and the energy-loss function *L*(*ω*) for the double perovskite Ba_2_InTaO_6_ are tabulated in Table 3.

**Table 3:** Calculated dielectric constant *ε*_1_(*ω*), absorption coefficient *α*(*ω*), refractive index *n*(*ω*) extinction coefficient *k*(*ω*) and reflectivity *R*(*ω*) of the real part of optical conductivity Re[*σ*(*ω*)] and the energy-loss function *L*(*ω*) of Ba_2_InTaO_6_ (in arbitrary unit)

Optical properties		Ba_2_InTaO_6_
GGA	*ε*_1_(0)	4.05
	*α*(*ω*)	4.35
	*n*(0)	2.01
	*k*(*ω*)	1.67
	*R*(0)	0.11
	*σ*(*ω*) (Ω cm)^−1^	6630.15
mBJ	*ε*_1_(0)	2.94
	*α*(*ω*) (eV)	5.56
	*n*(0)	1.71
	*k*(*ω*)	1.51
	*R*(0)	0.07
	*σ*(*ω*) (Ω cm)^−1^	6378.86

### 3.4 Thermoelectric properties

To investigate the transport of the belongings of the double perovskite oxide Ba_2_InTaO_6_, we have employed semi-classical Boltzmann theory and the rigid band approach as implemented in the BoltzTraP code^44^ interface with the Wien2K program. It peruses from these approaches that the dependence of the conductivity on transport distribution is:
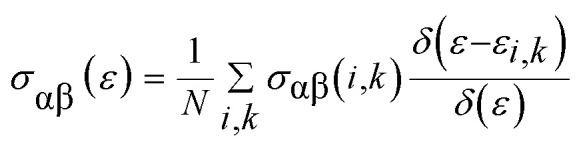


The *k*-dependent transport tensor is given as:*σ*_α,β_(*i*, *k*) = *e*^2^*τ*_*i*,*k*_*ν*_α_(*i*, *k*)*ν*_β_(*i*, *k*)

In the above equation, *i*, *k* and *τ*, *ν*_α_(*i*, *k*) and *e* express the band index, wave vector, relaxation time, the group velocities and the electron charge, respectively.
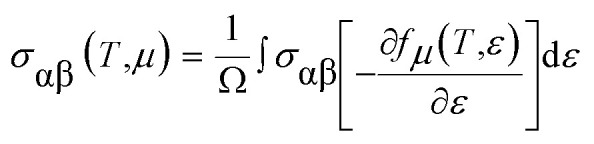


By integrating the transport distribution over energy, the electrical conductivity can be expressed as a function of temperature, *T*, and also the chemical potential, *μ*, *via* the subsequent equations:
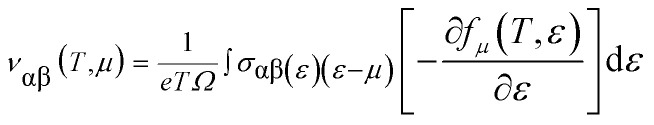


The complexity of the carrier scattering mechanisms in the solid arises from the relaxation time considered as the energy-independent constant. For evaluating the electrical transport properties, the above approach has been already tested on distinct compounds shown to be a reasonable approximation.

**Fig. 5:**
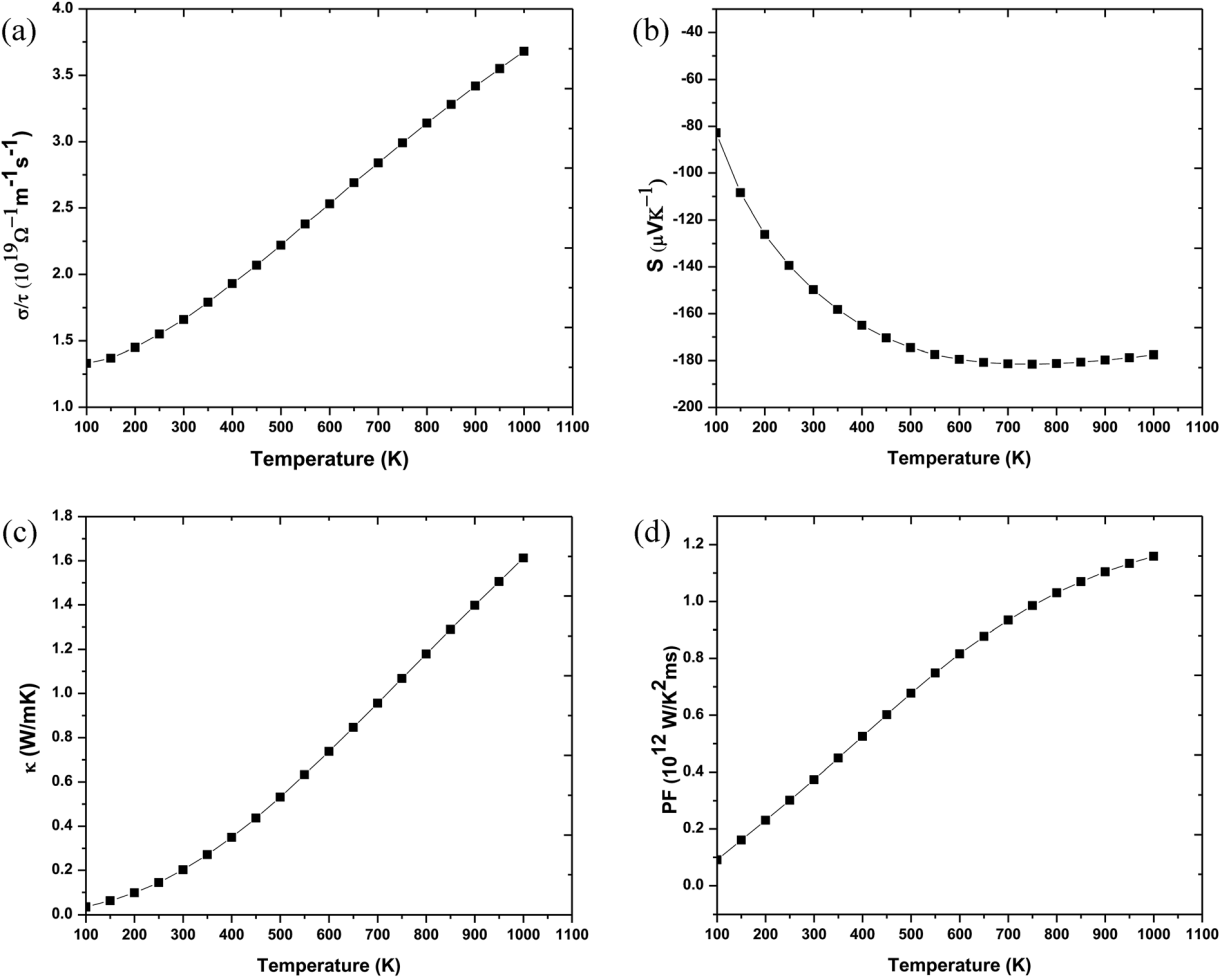
(a) Variation of electrical conductivity (*σ*/*τ*) as a function of temperature for Ba_2_InTaO_6_ compound. (b) Temperature-dependent Seebeck coefficient (*S*) as a function of temperature for the Ba_2_InTaO_6_ compound. (c) Temperature-dependent electronic thermal conductivity (*S*) as a function of temperature for the Ba_2_InTaO_6_ compound. (d) Calculated power factor (PF) as a function of temperature for the Ba_2_InTaO_6_ compound.

For a material to have efficient thermoelectric properties, it is mandatory to have high electrical conductivity, a large Seebeck coefficient and low thermal conductivity. Perovskites have received great attention as thermoelectric materials in the recent past. The thermoelectric properties like electrical conductivity (*σ*/*τ*), where *τ* is the relaxation time, the Seebeck coefficient (*S*), electronic thermal conductivity (*κ*_e_/*τ*) and the power factor (PF) were calculated. Fig. 5(a) presents the temperature variation of electrical conductivity (*σ*/*τ*) for Ba_2_InTaO_6_. The value of *σ*/*τ* does not vary much up to room temperature; it later increases with increasing temperature above room temperature. The increasing character of *σ*/*τ* suggests that the material should behave as a semiconductor. The value of *σ*/*τ* was found to increase from a small value of 1.33 × 10^19^ (Ω^−1^ m^−1^ s^−1^) at 50 K to 3.75 × 10^19^ (Ω^−1^ m^−1^ s^−1^) at 1000 K. The Seebeck coefficient (*S*) for the compound as a function of temperature is depicted in Fig. 5(b). It was seen from the plot that the calculated values of the (*S*) is negative in the complete temperature range; therefore, it suggests the presence of electrons as charge carriers. The value of *S* decreases from −76 μV K^−1^ at 50 K to −180 μV K^−1^ at 700 K and then again starts to increase smoothly upon a further increase in temperature. Fig. 5(c) depicts the response of thermal conductivity within 100 K and 1000 K for Ba_2_InTaO_6_. The figure clearly presents the increasing nature of thermal conductivity with respect to a temperature similar to the variation of the electrical conductivity plot. With the increase of temperature the value of (*K*_e_/*τ*) increases progressively from a very low value of 0.03 × 10^15^Ω^−1^ m^−1^ s^−1^ at 100 K to 1.61 × 10^15^ Ω^−1^ m^−1^ s^−1^ at 1000 K. The power factor (PF) is one of the significant parameters for evaluating the efficiency of a material for thermoelectric applications; it is actually the product of the Seebeck coefficient and electrical conductivity. Fig. 5(d) shows the variation of the calculated power factor with the temperature, showing that the power factor increases from 0.09 × 10^12^ W K^−2^ m^−1^ s^−1^ at 100 K to 1.20 × 10^12^ W K^−2^ m^−1^ s^−1^ at 1000 K. The pleasant value of the power factor implied that the Ba_2_InTaO_6_ material could possibly be a convenient material for high-temperature thermoelectric applications.

### 3.5 Thermodynamic properties

**Fig. 6:**
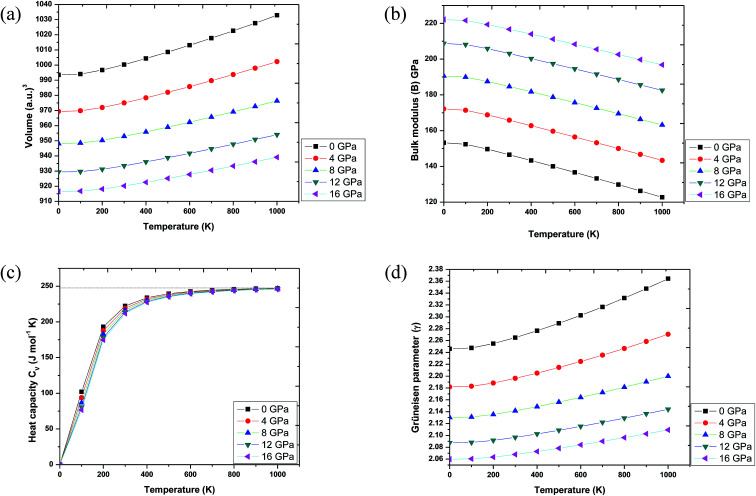
(a) Volume as a function of temperature and pressure for Ba_2_InTaO_6_. (b) Bulk modulus variation with temperature and pressure for Ba_2_InTaO_6_. (c) Specific heat capacity (*C*_v_) as a function of temperature and pressure for Ba_2_InTaO_6_. (d) Grüneisen parameter (*γ*) as a function of temperature and pressure for Ba_2_InTaO_6_.

In order to make Ba_2_InTaO_6_ a suitable material for device fabrications and other industrial applications, we have calculated various thermodynamic variables under temperature (0 K to 1000 K) and pressure (0 GPa to 16 GPa) using the quasi-harmonic Debye model.^41–43^ These thermodynamic variables like volume (*V*), bulk moduli (*B*), specific heat at constant volume (*C*_v_) and Grüneisen parameter (*γ*) were computed for Ba_2_InTaO_6_. Fig. 6(a) presents the variation in unit cell volume as a function of temperature and pressure. It can be clearly seen from the figure that cell volume increases with increasing temperature for a particular pressure value; on the other hand, it decreases with increasing pressure for a particular temperature value. Such behavior is quite general in solids, and one can understand that pressure compresses a solid, while temperature provides expansion. Fig. 6(b) shows the variation of bulk modulus under temperature and pressure. It is quite clear from the figure that the variation of the bulk modulus under temperature and pressure has the opposite effect, as noticed in the case of variation of volume under temperature and pressure. According to Fig. 6(b), the bulk modulus increases with increasing pressure and decreases with increasing temperature. One can understand the reason for such an increase or decrease in the B value with pressure and temperature that temperature reduces the hardness, while pressure increases it. Secondly, with increasing temperature, the unit cell volume increases and the interatomic distance also increases, while pressure has the reverse effect.

Further, the specific heat at a constant volume (*C*_V_), which provides information about the lattice vibration, phase transition and measures the motion of molecules, was calculated, and its variation with temperature and pressure is depicted in Fig. 6(c). From Fig. 6(c), one can see that the *C*_V_ increases rapidly under lower temperature values of 0 to 400 K. Above 400 K, a lethargic increase in *C*_V_ can be observed, which further becomes constant at a temperature of 700 K and reaches the famous Dulong–Petit limit.^60^ The value of *C*_V_ for Ba_2_InTaO_6_ at 300 K and 0 GPa was found to be 222.29 J mol^−1^ K^−1^. The calculated value of *C*_V_ under ambient conditions may serve as valuable data for experimental studies. The Grüneisen parameter (*γ*) expresses the anharmonicity in the crystal and was used to deduce the thermodynamic properties of the material under a high temperature and pressure. It also describes the temperature and pressure dependence of phonon frequencies or vibrational frequencies.^61^ We have, therefore, calculated the pressure and temperature dependence of *γ* for Ba_2_InTaO_6_ for the first time and is shown in Fig. 6(d). The value of *γ* increases slowly with increasing temperature and decreases with increasing pressure. The calculated value of *γ* under ambient conditions was found to be 2.26, which is in the order of the reported value of Ba_2_MgReO_6_.^62^

## 4. Conclusion

We summarized the present work by combining *ab initio* density functional theory, quasi-harmonic Debye model and the Post-DFT Boltztrap code used for the investigation of electronic structure as well as the elastic, mechanical, optical, thermoelectric and thermodynamic properties of the double perovskite Ba_2_InTaO_6_. The calculated electronic properties showed Ba_2_InTaO_6_ to be a semiconductor, and a direct band due to the lowest energy in the conduction region and the highest band within the valence region present on the same *X*−*X* symmetry point. There is a strong hybridization between the d states of Ba and Ta and the 2p states of oxygen, which mainly contribute to the electrical properties. By considering the elastic constant and mechanical calculations, the material was found to be mechanically stable and showed ductile and anisotropic nature. The large value of Young's modulus indicated that the material could serve as an important candidate for the manufacture of ultra-hard materials. Furthermore, by using the quasi-harmonic Debye model, the pressure and temperature dependence of thermodynamic parameters such as the volume, bulk moduli, heat capacity (*C*_v_) at constant volume and the Grüneisen parameter (*γ*) in the range 0–16 GPa and 0–1000 K are also projected and provide a possible future scope for suitable applications. The optical properties conjointly advocate the prominent utilization of Ba_2_InTaO_6_ in optical devices. It was found from the electronic band profile that the material had a wide band gap and was hence an important candidate for thermoelectric properties. Finally, thermoelectric properties like electrical conductivity (*σ*/*τ*), the Seebeck coefficient (*S*) and the power factor (PF) for Ba_2_InTaO_6_ were also reported for the first time. The pleasant value of the power factor implied that the material could also be used in thermoelectric devices.

## Conflicts of interest

There are no conflicts to declare.

## Supplementary Material
